# High reproducibility of a novel supported anterior drawer test for diagnosing ankle instability

**DOI:** 10.1186/s12891-023-06246-8

**Published:** 2023-02-27

**Authors:** Yasutaka Murahashi, Atsushi Teramoto, Katsunori Takahashi, Yohei Okada, Shinichiro Okimura, Rui Imamura, Makoto Kawai, Kota Watanabe, Toshihiko Yamashita

**Affiliations:** 1 Department of Orthopaedic Surgery, Sapporo Medical University School of Medicine, S-1, W-16, Chuo-Ku, Sapporo, 060-8543 Japan; 2 Division of Radiology and Nuclear Medicine, Sapporo Medical University Hospital, S-1, W-16, Chuo-Ku, Sapporo, 060-8543 Japan; 3 Division of Rehabilitation, Sapporo Medical University Hospital, S-1, W-16, Chuo-Ku, Sapporo, 060-8543 Japan; 4 Department of Physical Therapy, Sapporo Medical University School of Health Sciences, S-1, W-17, Chuo-Ku, Sapporo, 060-8556 Japan

**Keywords:** Ankle sprains, Ankle instability, Anterior talofibular ligament, Anterior drawer test, Capacitance-type sensor device

## Abstract

**Background:**

The manual traditional anterior drawer test (ADT) is essential for deciding the treatment for chronic ankle instability, but it has been shown to have a comparatively low reproducibility and accuracy, especially in less experienced hands. To clarify the inter-examiner reproducibility, we compared the actual distance of anterior translation between junior and senior examiners in ADT. We also evaluated the diagnostic abilities of traditional ADT, and a novel modified ADT (supported ADT).

**Methods:**

Thirty ankles were included in this study, and ankle instability was defined using stress radiography. All subjects underwent two methods of manual ADT by junior and senior examiners, and ankle instability was judged in a blinded fashion. The anterior drawer distance was calculated from the lengthening measured using a capacitance-type sensor device.

**Results:**

The degree of anterior translation determined by the junior examiner was significantly lower than that determined by the senior examiner when traditional ADT was performed (3.3 vs. 4.5 mm,* P* = 0.016), but there was no significant difference in anterior translation between the two examiners when supported ADT was performed (4.6 vs. 4.1 mm, *P* = 0.168). The inter-examiner reliability of supported ADT was higher than that of traditional ADT. For the junior examiner, the diagnostic accuracy of supported ADT was higher than that of traditional ADT (sensitivity, 0.40 vs. 0.80; specificity, 0.75 vs. 0.80).

**Conclusion:**

Supported ADT may have the advantage of being a simple manual test of ankle instability with less error between examiners.

## Background

Ankle sprains involving the lateral ligament are the most frequently occurring injuries in sports and physical activities [1, 2]. Most of them are treated conservatively, but in some cases, there may be residual symptoms, such as pain and instability during exercise and daily activity, which may progress to chronic ankle instability [3–5]. Chronic ankle instability is not only a residual symptom, but can also accelerate the development of osteoarthritis over a long period of time. While physical therapy may be attempted for patients with chronic ankle instability, structural mechanical dysfunction may be compensated for by supportive bracing, taping or operative treatment, such as ligament repair and reconstruction. Therefore, it is essential to identify the individual cause of ankle instability, and quantitative assessment of structural mechanical dysfunction is mandatory. Recent studies have demonstrated the quantitative evaluation of lateral ligament injury and ankle instability using MRI and stress ultrasonography [6–10]. Manual physical examination, called the anterior drawer test (ADT), is also an essential evaluation in making treatment decisions because of its convenience, cost, and dynamic nature [10, 11]. However, it has been pointed out that this procedure can be subjective, lacks reproducibility, and can be difficult, especially for less experienced clinicians [12, 13].

To improve diagnostic accuracy, several studies on the optimal ankle joint angle and the magnitude and direction of the force applied in performing the anterior drawer test have been reported [13–17]. Allowing for the internal rotation of the ankle during traditional ADT may make it easy for the examiner to detect more subtle degrees of ankle instability and result in higher diagnostic sensitivity [13, 16]. Li et al. recently showed that the reverse anterolateral drawer test method is more sensitive and accurate in diagnosing chronic anterior talofibular ligament injuries than traditional ADT [10]. Although various techniques and methods have been reported to improve diagnostic accuracy, little consideration has been given to test reproducibility.

The reason for the comparatively low reproducibility and accuracy of traditional ADT, especially in less experienced hands, may be that the test requires two hands for manipulation and relative hand movements make it difficult to perceive ankle instability [10, 12, 13]. It is difficult to quantify and categorize the magnitude of the instability, which can only be judged by the perception of the hand movement. Additionally, ADT involves both the talocrural and subtalar joints. Furthermore, relieving gastrocnemius muscle tonus is not easy for junior examiners with less experience. Hence, we evaluated the usefulness of a novel modified ADT, called supported ADT, in which one hand of the examiner is supported while stabilizing the distal tibia. We also measured the actual distance of anterior translation in the manual test to clarify the inter-rater reliability of traditional and supported ADTs. The objective of this study was to compare the reproducibility and accuracy of the two ADTs and to determine the usefulness of supported ADT. We hypothesize that supported ADT has higher reproducibility of dynamic anterior drawer displacement between junior and senior examiners and higher diagnostic ability than traditional ADT in the diagnosis of ankle instability, particularly by junior examiners.

## Methods

### Subjects

The subjects were 30 ankles (24 patients) with or without a history of ankle sprain who complained of instability or pain. First, all patients underwent stress radiography, and ankle instability was defined using stress radiography. Second, all patients underwent manual ADT using two methods; the procedure was performed by a junior resident doctor (junior examiner: orthopedic surgeon with 2 years of experience) and senior foot and ankle surgeon (senior examiner: orthopedic surgeon with 23 years of experience) who were blinded.

#### Stress radiography

**Fig. 1:**
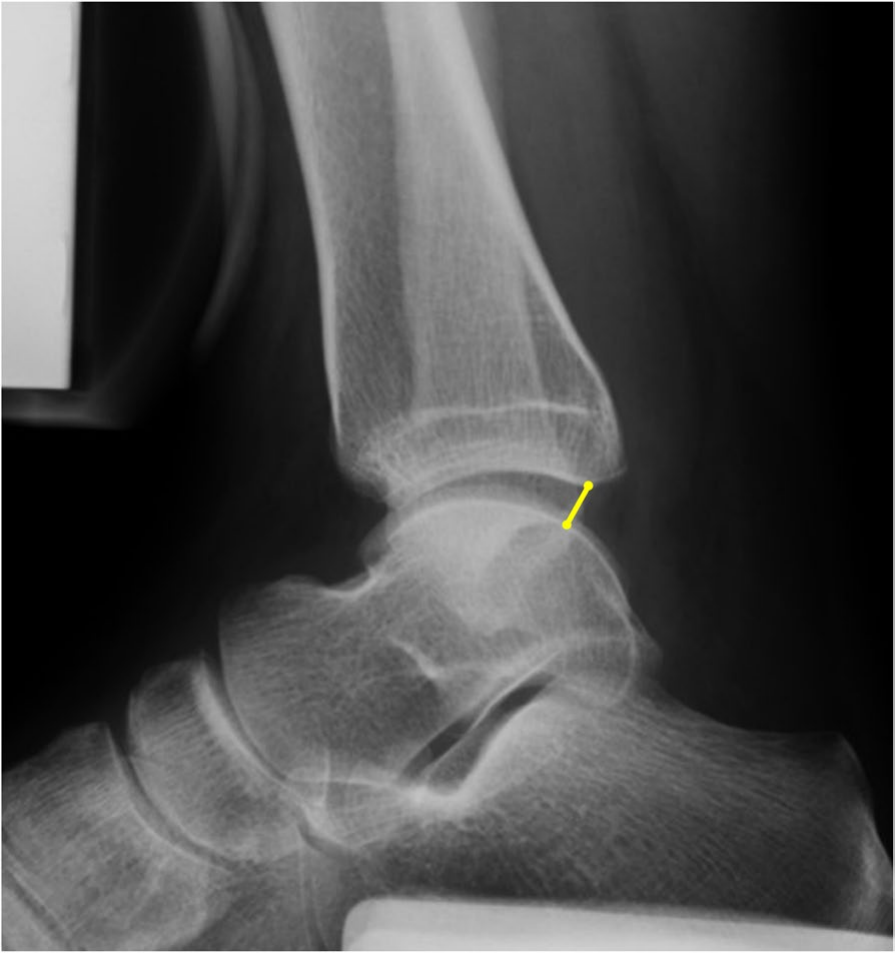
Radiographic measurement of anterior talar translation using TELOS stress device. Anterior talar translation is the distance between the posterior lip of the tibial articular surface and the nearest point of the talar articular surface (yellow line)

Lateral stress radiographs of the ankles were taken in two projections using the Telos stress device (Telos, Griesheim, Germany). Anterior drawer stress was applied with a magnitude of 150 N of posteriorly directed force to the tibia, which allowed the visualization of changes in the neutral position of the joint ends in the ankle joint under constant stress. The distance of anterior talar translation was measured from the posterior lip of the tibial plafond to the nearest articular surface of the talus (Fig. 1). In comparison with the unstressed position, anterior subluxation of the talus of more than 3.5 mm was considered to be indicative of ankle instability [18, 19].

#### Manual anterior drawer test

**Fig. 2:**
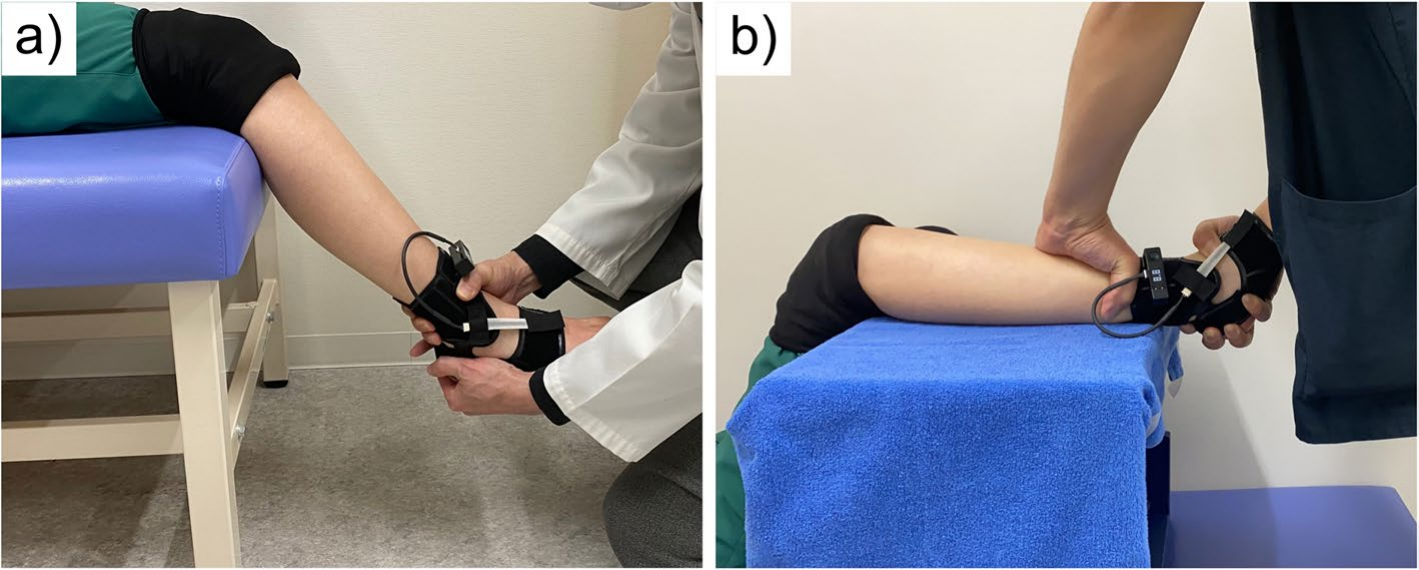
Manual anterior drawer tests with capacitance-type sensor device. The traditional anterior drawer test is shown in **a**. The patient rested in a sitting position, with the ankle joint in a slightly flexed position. A supported anterior drawer test was conducted with the lower leg placed on the board with the subject in supine position and the examiner stabilized the lower leg on the board **b**

To perform traditional ADT, the patient rested in a sitting position, with the ankle joint in a slightly flexed position. (Fig. 2a). The examiner pulled the calcaneus of the subject forward with one hand, while pushing the tibia backward with the other hand to reverse the direction of the forces. The examiner evaluated ankle instability using the sensation of the relative motion between the two hands.

Supported ADT was performed in the supine position with the lower leg placed on a board with the hip and knee joints in 90-degree flexion and the ankle joint in 10- to 20-degree plantar flexion (Fig. 2b). The examiner stabilized the lower leg on the board, pulled the calcaneus forward with one hand, and judged the ankle instability by the sensation of the movement of one hand.

The two methods of ADT were performed by a junior and senior examiner. The examiner graded the amount of posterior displacement of the talus using manual ADT, with a grade of 1 as stable joint, 2 as partially unstable, and 3 as completely unstable. Grades 2 and above were considered positive for excessive instability, whereas grade 1 was considered negative or normal [6].

#### Anterior drawer displacement measured by the sensor during manual anterior drawer test

To clarify the actual distance of anterior displacement in the manual ADT, we evaluated anterior translation measurements using a capacitance-type sensor device, as previously described [20]. The capacitance-type sensor device has high reproducibility and correlated with stress radiography measurements in patients with ankle instability, and is useful for a quantitative evaluation of anterior drawer laxity [20]. Briefly, braces hold the foot and ankle, and allow freedom of movement between them. The sensor was fixed with a snap button to the lateral malleolus and talar part of the brace, and the anterior drawer distance was calculated from the lengthening measured by the sensor. Traditional and supported ADTs were also performed by junior and senior examiners, as mentioned above, to measure the anterior drawer displacement. ADT was conducted three times by each of the two examiners. The anterior drawer distance was calculated from the lengthening of the sensor (mm). Data were expressed as the average of three trials in each participant.

### Statistical analysis

Statistical significance was evaluated using the Levene’s test for the analysis of equality of variances and the Student’s t-test for comparison using EZR, a graphical user interface for R (The R Foundation for Statistical Computing, Vienna, Austria) [21]. Pearson’s correlation coefficient was used to determine the linear relationship between the anterior drawer displacement of manual ADT between the senior and junior examiners. To assess the diagnostic performance of the tests, the sensitivity, specificity, and positive and negative predictive values were calculated for each examination. The correlation between junior and senior examiners in terms of the accuracy of each physical examination was assessed using the kappa statistic. To assess the relationship between the anterior drawer displacement of the manual ADT and the stress radiography measurements, Bland–Altman plots were used to provide a visual representation of the degree of agreement. Statistical significance was set at *P* less than 0.05.

### Ethics and registration

This study was conducted in accordance with the principles of the Declaration of Helsinki and Good Clinical Practice guidelines. The study was approved by the Institutional Review Board for Clinical Research of our hospital (reference number 29–242), and informed consent was obtained from all study participants after providing patients with written and oral information before inclusion in the study.

## Results

Table 1 shows the demographic data and the anterior talar translation distance on stress radiography. The male-to-female ratio of patients was approximately 1:3. The mean age was 32.0 (range, 23–38) years, and body mass index (BMI) was 22.4 (range, 17.1–30.0) kg/m2. Ankle instability was defined in 10 cases by anterior drawer stress radiography.

**Table 1:** Patient demographics and distance of anterior talar translation (ATT)

Sex	Male	6 (8 feet)	
	Female	18 (22 feet)	
Age (y), mean [range]		32.0	[23–38]
Height, cm [range]		171.1	[154–185]
Body weight, kg [range]		65.9	[46–92]
Side	Rt	20	
	Lt	10	
ATT distance	< 3.5 mm	20	
	≥ 3.5 mm	10	

Data are presented as frequencies, unless otherwise indicated.

*ATT*, anterior talar translation of stress radiography

In the manual ADTs, the junior examiner diagnosed 9 cases with instability by traditional ADT and 12 cases with instability by supported ADT. The senior examiner diagnosed 16 cases with instability by traditional ADT and 10 cases with instability by supported ADT. For the junior examiner, the diagnostic accuracy of the manual tests for detecting ankle instability defined by stress radiography had a sensitivity of 0.4 and 0.8, with a specificity of 0.75 and 0.8 in traditional and supported ADTs, respectively (Table 2). For the senior examiner, the diagnostic accuracy had a sensitivity of 0.8 and 0.7 with a specificity of 0.6 and 0.85 in the traditional and supported ADTs, respectively. The kappa values of the inter-examiner reliability for detecting instability in the traditional ADT and supported ADT were 0.416 and 0.571, respectively.

**Table 2:** Diagnostic accuracy of two manual anterior drawer tests by the senior and junior examiners

		Sensitivity (95% CI)	Specificity (95% CI)	PPV (95% CI)	NPV (95% CI)	κ
Traditional ADT	Junior examiner	0.400 (0.122–0.738)	0.750 (0.509–0.913)	0.444 (0.137–0.788)	0.714 (0.478–0.887)	0.416 (0.096–0.735)
	Senior examiner	0.800 (0.444–0.975)	0.600 (0.361–0.809)	0.500 (0.247–0.753)	0.857 (0.572–0.982)	
Supported ADT	Junior examiner	0.800 (0.444–0.975)	0.800 (0.563–0.943)	0.667 (0.349–0.901)	0.889 (0.653–0.986)	0.571 (0.265–0.878)
	Senior examiner	0.700 (0.348–0.933)	0.850 (0.621–0.968)	0.850 (0.621–0.968)	0.722 (0.465–0.903)	

Data are presented as mean (range), unless otherwise noted

*ADT*, anterior drawer test, *CI* confidence interval, *PPV* positive predictive value, *NPV* negative predictive value

**Fig. 3:**
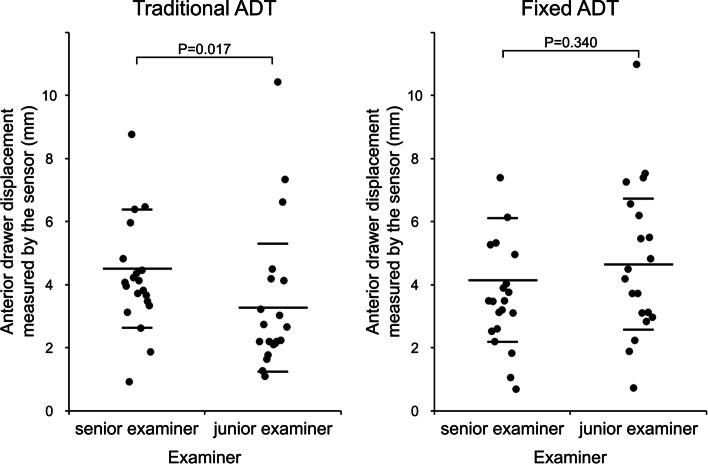
Difference in anterior drawer displacement between the senior and junior examiner. ADT, anterior drawer test

**Fig. 4:**
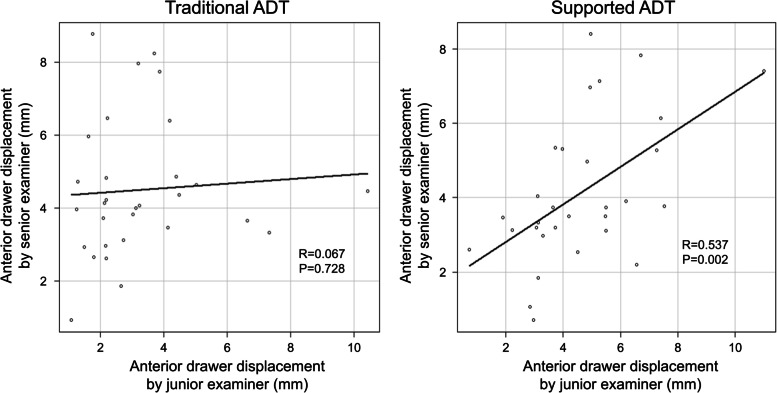
Correlation of anterior drawer displacement between the senior and junior examiners in the two anterior drawer tests. ADT, anterior drawer test

**Fig. 5:**
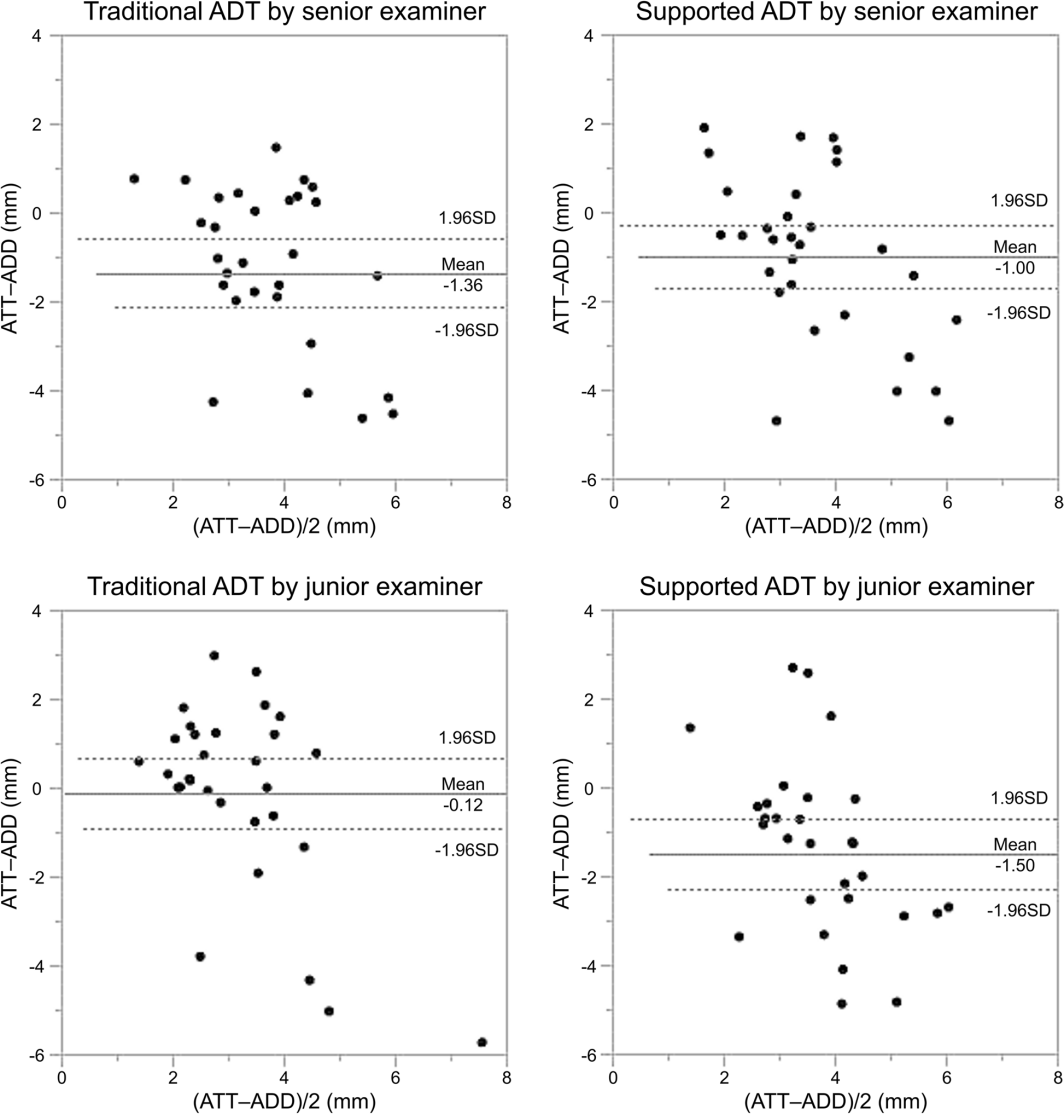
Bland–Altman plots of identical measurements by the XP and the ADT. ADD, anterior drawer displacement of the anterior drawer test with capacitance-type sensor device; ATT, anterior talar translation of stress radiography

Equal variances of the anterior translation distance using the capacitance-type sensor device were assumed (traditional ADT, *P* = 0.695; supported ADT, *P* = 0.742) when using the Levene’s test for the analysis of equality of variances. The distance of anterior translation determined by the junior examiner using the capacitance-type sensor device was significantly lower than that of the senior examiner in traditional ADT (3.26 ± 2.03 vs. 4.5 ± 1.88, *P* = 0.017), but with the numbers available, there was no significant difference in anterior translation between the examiners in supported ADT (4.64 ± 2.08 vs. 4.14 ± 1.96, *P* = 0.340) (Fig. 3). The correlation coefficient of anterior drawer displacement between examiners was highly significant in supported ADT (*R* = 0.537; *P* = 0.002; 95% CI, 0.22–0.75), and with the numbers available, there was no significant correlation in traditional ADT (*R* = 0.067; *P* = 0.728; 95% CI, -0.30–0.42) (Fig. 4). Finally, Bland–Altman plots were used to assess the mean difference and the degree of agreement between the measurements of ADT and stress radiography. The Bland–Altman plots showed a lower level of agreement in both traditional ADT and novel ADT (Fig. 5).

## Discussion

We evaluated the anterior drawer distance in the ADTs between examiners using a capacitance-type sensor device and determined the usefulness of supported ADT. In traditional ADT, the measurements of anterior drawer displacement by the junior examiner were significantly lower than those by the senior examiner, and the sensitivity of traditional ADT performed by the junior examiner was relatively low. However, the distances of anterior displacement between examiners were similar in the supported ADT, and the test was sensitive, even when performed by the junior examiner.

Several studies have assessed ankle instability using stress radiography and/or ultrasonography. Tarczyńska et al. evaluated 30 patients with chronic ankle instability treated surgically or nonoperatively and showed that stress radiography is a reliable and objective imaging tool for diagnosing ankle instability [1]. Lee et al. suggested that the anterior talofibular ligament length on stress ultrasound could be useful for diagnosing chronic ankle instability in addition to the manual anterior drawer test and stress radiography [9]. However, these diagnostic methods are limited by the availability of the instruments and potential errors from radiography and ultrasonography [13]. Most surgeons rely on the manual anterior drawer test to determine treatment.

Manual ADT is essential in clinical settings, but requires experience to perfect the technique, and low sensitivity of the test may be due to technical variation between individual examiners, especially with less experienced examiners [12, 13]. In the ADT procedure, axial load increases the stability of the ankle joint complex and results in a reduction in the sensitivity of ADT in detecting mechanical ankle instability [22]. In a biomechanical cadaveric study, Tohyama et al. indicated that ADT with an ankle positioned between 10 and 20 degrees of plantar flexion produced the most laxity and least stiffness [17]. They also suggested that an excessive magnitude of anterior load is not necessary to detect disruption of the anterior talofibular ligament during the anterior drawer test [23]. In a study using ultrasonography, ADT of the anterior talofibular ligament-deficient ankle at 20 degrees of plantar flexion induced greater length changes in the anterior talofibular ligament than ADT with the ankle in neutral position [14]. As mentioned above, manual ADT requires certain technical points to be proficient in the procedure. Most studies refer only to the modified technique and diagnostic accuracy of ADT, and there are few reports on verifying its reproducibility or inter-rater reliability. The reason for this is that quantitative measurements of anterior translation in manual ADT are difficult in vivo. In the current study, the distance of anterior displacement between examiners was quantitatively measured and its reproducibility was evaluated using a capacitance-type sensor device. In this study, supported ADT had high diagnostic accuracy, even with junior examiners, and the distance of anterior translation was similar to that determined by the senior examiner. This may be due to the fact that placing the lower leg on the board with the hip and knee flexed at 90 degrees with the subject in supine position makes it easy for them to relax and relieves gastrocnemius muscle tonus. Kovaleski et al. indicated that ADT with the knee positioned at 90-degree flexion produces more laxity than that with the knee at 0-degree flexion, which may be accounted for by increased tension of the gastrocnemius-Achilles tendon complex when the knee is extended, increasing the ankle complex stiffness during ADT [15]. Furthermore, the examiners perform traditional ADT by applying an anteriorly directed force to the calcaneus with a backward push on the tibia, and evaluate the ankle joint laxity by two-hand manipulation [18]. Thus, examiners need to evaluate ankle instability by the relative movements of both hands, and complex movements and subtle sensations are required, resulting in low sensitivity and reproducibility, especially with less experience. In supported ADT, examiners may decrease the sensation of relative hand movements by stabilizing the distal tibia with one hand, making it easier to diagnose ankle instability. Supported ADT is a particularly preferred method for evaluation of ankle instability by inexperienced examiners who are not foot specialists.

In a previous study using a capacitance-type sensor device, the anterior drawer distance of ADT measured by sensor were examined in cadavers [20]. The mean anterior drawer distance for the intact ankles was 3.7 ± 1.0 mm, and the distance for the ankle transected anterior talo-fibular ligament (ATFL) was 6.1 ± 1.6. In our study, the distance measured by the junior and senior examiners in traditional ADT was 3.26 ± 2.03 mm and 4.5 ± 1.88 mm, respectively. The mean values and standard deviations were relatively different in the previous study and the current study, which might result from differences between the cadavers and the patients and might be related to the presence or absence of muscle tonus in the subjects during the examination. In Bland–Altman plots of stress radiography results vs. manual ADT results, there was low reliability and the mean lengths of the manual ADT were greater than those of stress radiography. The stress radiography was performed with a single force in the anterior direction, and the values were measured by a single direction. On the other hand, manual ADT with sensor was performed with an anterior force during internal rotation, and the values were measured by a three-dimensional direction. This difference might account for the discrepancies in the measured values of the two examination methods.

This study has several limitations. First, the number of cases in our study was relatively small, which may have affected the statistical analysis. Second, there was no difference in the diagnostic accuracy between traditional and supported ADTs for senior examiners, and further larger studies are warranted. Finally, the diagnostic accuracy was evaluated based on the instability defined by stress radiography in this study. Several reports previously pointed out the low accuracy and reproducibility of stress radiography in diagnosing ankle instability [24, 25]. However, since stress radiographs have been called into question, there is no established gold standard for quantifying ankle instability [26]. Many novel diagnostic methods of ankle instability have recently been reported, such as stress sonography, stress MRI, the score of the perceived ankle instability (including the Cumberland Ankle Instability Tool), and functional impairment evaluation (including stabilometry) [6, 14, 24, 25, 27, 28]. In the future, it is necessary to evaluate the validity of the novel ADT by establishing an objective definition of mechanical ankle stability. Despite these limitations, the distance of anterior translation by the junior examiner was significantly lower than that by the senior examiner in traditional ADT and the distance of anterior translation is similar between examiners in supported ADT. To the best of our knowledge, there are no reports documenting a comparison of the actual distances of anterior displacement in manual anterior drawer test in vivo.

## Conclusions

Supported anterior drawer test has a higher inter-rater reliability than the traditional procedure, and a similar amount of anterior translation was observed between junior and senior examiners when it was performed. Supported ADT is a simple manual examination, and may be useful for the evaluation of ankle instability as a method with less error between examiners.

## Data Availability

The datasets generated and/or analysed during the current study are not publicly available due to limitations of ethical approval involving the patient data and anonymity but are available from the corresponding author on reasonable request.

## Abbreviations

MRI: Magnetic Resonance Imaging
ADT: Anterior drawer test
